# Fabrication and Characterization of Microcellular Polyurethane Sisal Biocomposites

**DOI:** 10.3390/molecules24244585

**Published:** 2019-12-14

**Authors:** S.M.S. Abdel-Hamid, O.A. Al-Qabandi, Elminshawy. N.A.S., M. Bassyouni, M.S. Zoromba, M.H. Abdel-Aziz, H. Mira, Elhenawy Y.

**Affiliations:** 1 Department of Chemical Engineering, the Egyptian Academy for Engineering and Advanced Technology, Affiliated to Ministry of Military Production, Al Salam city 3056, Egypt; 2 EQUATE Petrochemicals Company, P.O. Box 91717, Ahmadi 61008, Kuwait; dr.o.alqabandi@gmail.com; 3 Department of Mechanical Engineering, Faculty of Engineering, Port Said University, Port Fouad 42526, Egyptdr_yasser@eng.psu.edu.eg (E.Y.); 4 Department of Chemical Engineering, Faculty of Engineering, Port Said University, Port Said 42526, Egypt; 5 Materials Science Program, University of Science and Technology, Zewail City of Science and Technology, October Gardens, 6th of October, Giza 12578, Egypt; 6 Chemical and Materials Engineering Department, King Abdulaziz University, Rabigh 21911, Saudi Arabia; mohamedzoromba@yahoo.com or; 7 Chemistry Department, Faculty of Science, Port Said University, Port-Said 42521, Egypt; 8 Chemical Engineering Department, Faculty of Engineering, Alexandria University, Alexandria 21526, Egypt; 9 Nuclear Materials Authority, Cairo 11381, Egypt; hamedmira@yahoo.com

**Keywords:** sisal, polyurethane, biocomposites, viscoelasticity, thermal expansion, CES

## Abstract

In this study, microcellular polyurethane (PU)-natural fiber (NF) biocomposites were fabricated. Polyurethanes based on castor oil and PMDI were synthesized with varying volume ratios of sisal fiber. The effect of natural fiber treatment using water and alkaline solution (1.5% NaOH) and load effect were investigated. Biocomposites were mechanically and physically investigated using tensile, viscoelasticity, and water absorption tests. The interfacial adhesion between PU and sisal fiber was studied using SEM. Short NF loads (3%) showed a significant improvement in the mechanical properties of the PU-sisal composite such as modulus of elasticity, yield and tensile strength up to 133%, 14.35 % and 36.7% respectively. Viscoelastic measurements showed that the composites exhibit an elastic trend as the real compliance (J’) values were higher than those of the imaginary compliance (J’’). Increasing NF loads resulted in a decrease of J’. Applying variable temperatures (120–80 °C) caused an increase in the stiffness at different frequencies.

## 1. Introduction

Over the past few decades, polymers have substituted for several types of metals in different applications. This can be attributed to the advantages polymers and polymer composites offer over conventional materials [1,2,3]. For example, bending restrictors (cable support products) were originally made of steel, however, steel tends to be heavy and expensive, and it will corrode. Nowadays, bending restrictors manufactured from polyurethane have largely solved these problems. Polymers have easy processing, high productivity, and reduced costs. In most applications, polymers are modified using additives and fibers to meet different mechanical, electrical and thermal requirements [4,5,6,7]. Polymer composites are a kind of customized material, designed from a combination of two or more material phases, including matrix and dispersed phases. Multiphase solid materials are selected for their superior mechanical, electrical or thermal properties, which are substantially higher than those of a single material. Fiber-reinforced plastics provide advantages over other conventional materials when specific properties are compared [8,9,10]. These composites find applications in diverse fields, from appliances to spacecraft.

Polyurethane is one of the polymers that play an important role in our life [11,12,13]. It plays a part in many industries; from footwear to ship-building, and it can be found in a constantly increasing variety of forms [14,15,16,17]. Automotive companies have focused recently on several polymer biocomposite materials in order to obtain materials with vibration damping and sound absorption properties in addition to light weight for reduced fuel consumption [18]. Biocomposites are a potential candidate to replace the glass-fiber sheet molding compound (GF-SMC) used as automotive panels.

Polyurethane products are used in several applications, such as rigid and flexible foams, coatings, elastomers, fiber and fabrics, adhesives, sealants, and composites. Polyurethane-based composites are widely used in automotive parts such as caravans, bumpers, covers, flaps, rear ends, hoods, and roof modules for commercial vehicles due to their low-density, superior mechanical properties and easy processability. PUs are mainly synthesized by the reactions of isocyanate (NCO) with: (i) polyols to yield polyurethanes; (ii) amines to form polyureas; (3) water to give polyurea and carbon dioxide which are considered blowing agents in PU foams; (4) urethanes, forming biuret and allophanate cross-linking groups. High resilience flexible polyurethane foams are synthesized using polymeric polyols. And other ingredients like castor oil, hydroxyl-terminated polybutadiene (HTPB), inter alia. Polyols with molecular weight (MWt) in the range of 1000–6000 and functionality values (the average number of –OH groups) between 1.8 and 3.0 are usually used to fabricate flexible PU foams and elastomers. High cross-linked rigid PUs are synthesized using short chain polyols (MWt < 1000) with high functionality (3–12) [19].

**Figure 1:**
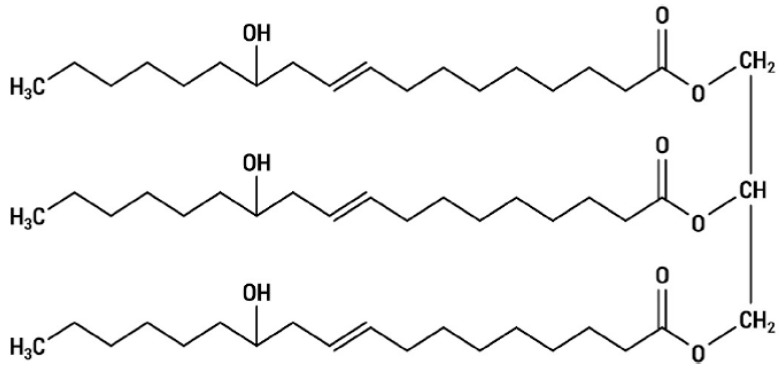
Structure of ricinoleic acid triglyceride.

Vegetable oils such as soybean and castor oil are considered renewable materials in manufacturing polyurethanes [20,21,22]. The total fatty acids in castor oil are constituted in 90% by ricinoleic acid. Triglycerides derived from the ricinoleic acid are obtained by pressing or solid-liquid extraction of the seed of the plant *Ricinus communis*. Compared with other vegetable oils, castor oil has singular chemical characteristics due to the presence of ricinoleic acid. Figure 1 shows the chemical structure of ricinoleic acid triglyceride. It has three types of reactive functional group: the hydroxyl group which is attached to the 12th carbon; unsaturation (between the 9th and 10th carbon); and the carbonyl group, found on the 1st carbon. These features characterize the triglyceride as a trifunctional polyester [23,24,25].

Polyurethane foams are commonly synthesized by the reaction of isocyanates with polyols in the presence of water as a blowing agent, resulting in formation of carbon dioxide (CO_2_), which can give a closed-cell or open-cell internal foam structure [26,27,28]. Polyurethane foams can be used for several applications, particularly when high energy absorption capabilities are required. The structural response of polyurethane foam mainly depends on its cell size and shape, open or closed-cell structure and foam density [29,30]. Owing to its viscoelastic properties, polyurethane foam exhibits a strain-rate dependent behavior [28,31]. Viscoelastic behavior is distinctly different from elastic models. Elastic materials can usually store up to 100% energy in compression and tension, whereas some of this energy is dissipated as heat in viscoelastic materials. Polyurethane composite foams have a wide spectrum of applications. Recently, they have been used mainly in the aerospace and automotive industries, as electromagnetic interference shielding, as oil absorbents, sensors, in fire proofing, shape memory products, biomedical materials and in radar absorbing materials.

Natural fibers have lately been widely introduced for the reinforcement of composites for several environmental and economic reasons [32,33]. Besides being cost effective, natural fibers are biodegradable, nonabrasive and readily available. Their light density is also considered a major advantage, especially in the car industry, as low weight means fuel savings. The feasibility and value of the different natural fibers depends on their end-use markets and costs of extraction. These properties make natural fibers able to compete with glass fibers in composite materials [34,35,36]. However, there are some disadvantages that restrict the use of natural fibers in industry, such as incompatibility with specific polymeric matrices, formation of aggregates while processing, and their poor resistance to moisture [37,38,39]. To control these drawbacks, natural fibers are often treated with suitable chemicals/methods, e.g., silane, graft copolymerization, isocyanate, mercerization, acetylation, benzyl compounds and acrylamide [40].

In this study, experimental work was conducted to investigate the influence of sisal fiber loadings on polyurethane foam. The effect of fiber chemical treatment using diluted sodium hydroxide on the mechanical, viscoelastic and water up-take properties of the resulting microcellular polyurethane/sisal biocomposites were studied. A predictive model, using the Cambridge Engineering Selector (CES) program, was applied to study the mechanical and thermal properties of polyurethane foams at different sisal fiber loadings. The main objectives of this study are to identify, develop and test biocomposite material (sisal fiber)-based PU for superior mechanical and physical properties, improving the fiber-matrix interaction, interfacial adhesion and validation of their properties using CES program for several applications such as car bumpers and thermal insulation.

## 2. Results and Discussion

The mechanical properties of M-C polyurethane sisal biocomposites are characterized using a universal tensile machine. It was found that tensile strength increased with increasing fiber loading. Untreated sisal fiber showed a remarkable improvement in the tensile and yield strength of M-C PU biocomposites. Surface morphology results showed a good fiber-matrix interfacial bonding which is in a good agreement with the mechanical tests. The thermal expansion of PU foam was enhanced in the presence of sisal fiber. The effect of sisal fiber loadings and temperature on the viscoelastic properties showed that fiber loading leads to a decrease in real compliance.

### 2.1. Tensile Test

**Figure 2:**
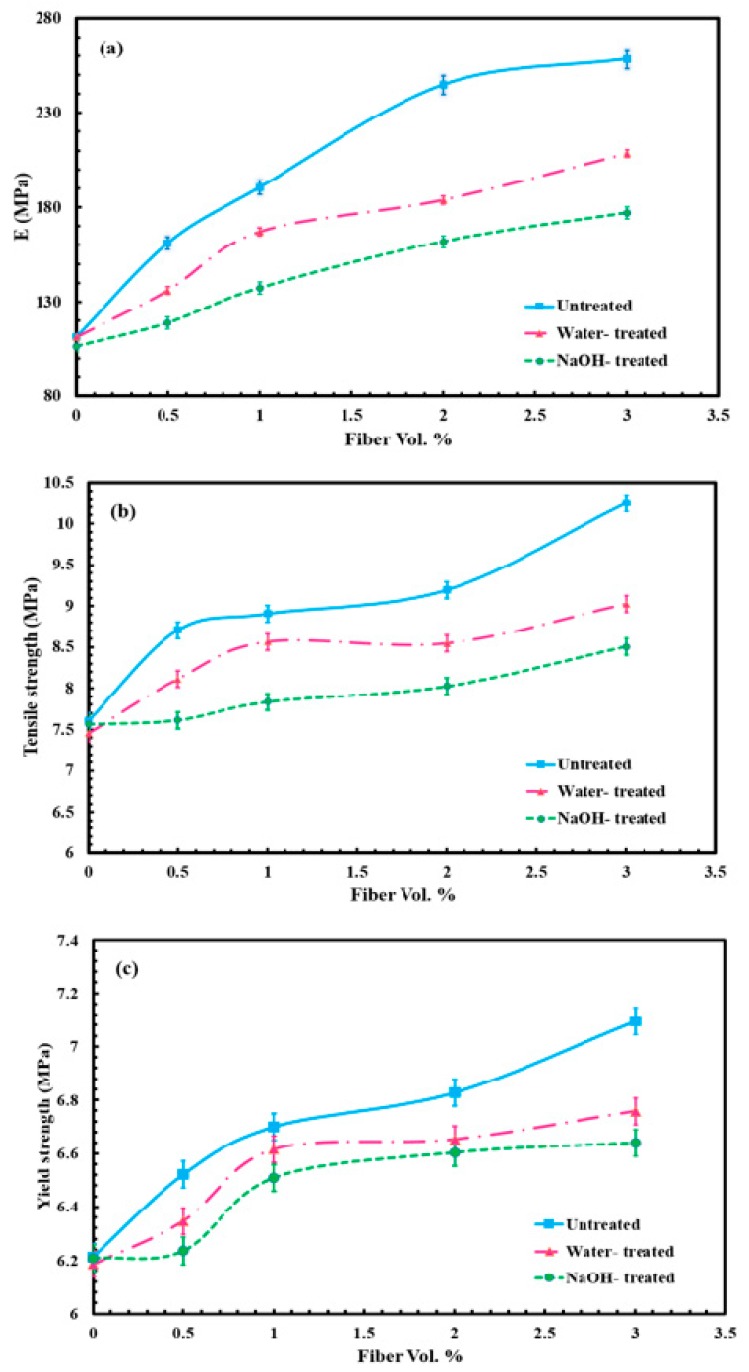
Mechanical properties of M-C polyurethane biocomposites. (**a**) Modulus of elasticity, (**b**) tensile strength and (**c**) yield strength.

Figure 2a–c show that the elastic modulus, tensile strength and the yield strength are increased for all treated and untreated sisal fibers, with increased fiber loadings in microcellular (M-C) polyurethane biocomposites. It was found that at the maximum loading (3 vol%), the elastic modulus increased by 133.3, 89.5 and 59.17% for untreated, water and NaOH-treated specimens respectively. The numerical simulation of the CES program has been applied to study the effect of untreated sisal loadings on the yield strength of microcellular polyurethane. 

**Figure 3:**
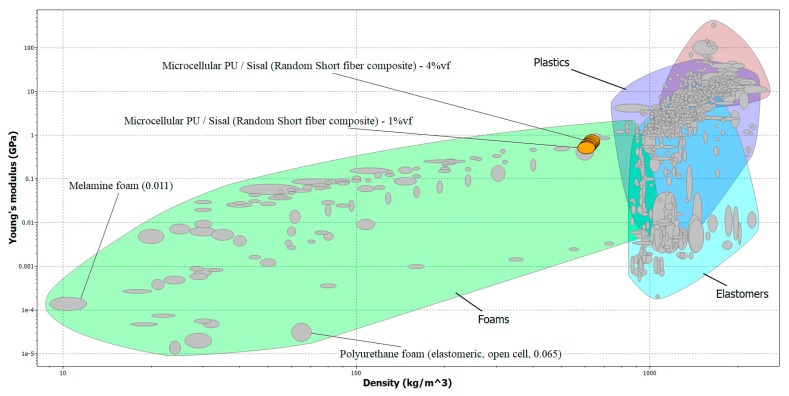
Yield strength of untreated sisal/ M-C Polyurethane biocomposites using CES program.

Figure 3 shows that the yield strength of untreated sisal/M-C polyurethane increased from 5.1 to 11.2 MPa when the untreated sisal fiber loading was increased from 0 to 4% by volume. All mechanical simulation results using the CES program are listed in Table 1. It was found that the experimental results are similar to the CES-program results using fiber loadings in the range of 0 to 2.5 vol%. Yield strength increased by 14.35, 9.1 and 6.6% for the untreated, water treated and NaOH treated sisal fiber composites respectively. NaOH treatment produced the lowest mechanical properties and this could be due to the attack on hydroxyl (OH) groups on the surface of fibers by NaOH [41]. In other polymers, this should enhance the interfacial adhesion with the natural fiber. However, for polyurethane, those OH groups react with the NCO groups of the diisocyanate and this reaction is responsible for the good adhesion of the fibers to the PU matrix. Hence, attacking the OH groups would weaken the interfacial bonds between the fiber and the PU matrix [42]. Water treatment may lead to the removal of some impurities that reduce the fiber surface roughness, and thus decrease its adhesion to the polymer matrix and consequently the mechanical properties. The linear thermal expansion coefficient α is the thermal strain per degree C [1,14]. 

**Figure 4:**
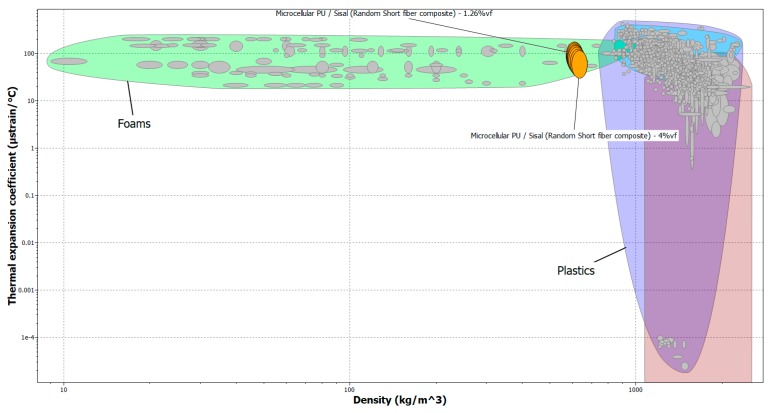
Thermal expansion coefficient of untreated sisal/ M-C Polyurethane biocomposites using CES program.

Figure 4 shows that the thermal expansion of M-C polyurethane can be decreased from 114 to 60 µstrain/°C in the presence of sisal fiber from 0 to 4 vol% respectively. 

This could be attributed to the lower thermal expansion coefficient of sisal fiber (22.5 µstrain/°C) compared with M-C polyurethane (114 µstrain/°C). Natural fiber, tightly packed in the polymer matrix, imposes a mechanical restraint on the expansion of the polyurethane chains during heating and thus helps decrease the overall thermal expansion coefficient of the biocomposites [42]. The incorporation of sisal fiber causes a remarkable reduction (47%) in the thermal expansion coefficient values when the sisal fiber loading content is 4 vol%. Hence, it seems that the use of sisal fiber at low content level vol% can effectively control thermal expansion behavior of M-C polyurethane.

### 2.2. Water Uptake

Water absorption depends on the polymer, natural fiber loadings, method of processing and environmental conditions. Natural fiber water absorption can be attributed to the presence of hydroxyl groups which uptake water through formation of hydrogen bonds [24].

**Figure 5:**
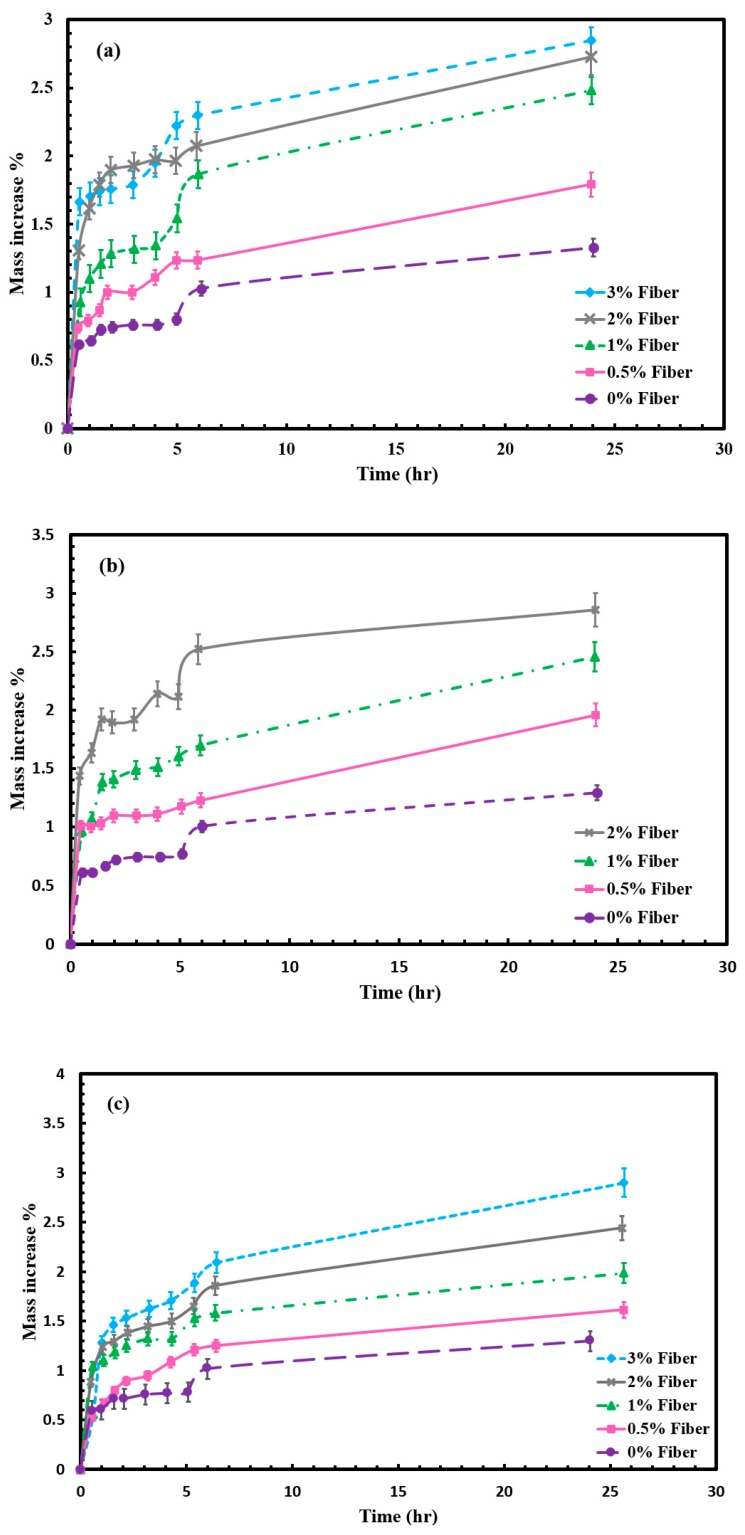
Water uptake for untreated and treated sisal/M-C polyurethane biocomposites. (**a**) Water treatment, (**b)** untreated sisal biocomposite and (**c**) alkali treatment.

Figure 5a–c show the variation of water absorption of the M-C polyurethane biocomposites with increasing fiber loading. All the composites with different fiber treatments exhibited an increase in mass with increasing soaking time in water. They also exhibited an increase in water absorption proportional to the fiber loadings. The water uptake property increases as the natural fiber loading increases and it will follow Fickian behavior. This phenomenon can be explained by considering the water absorption characteristics of natural fibers. When the polyurethane biocomposites are exposed to moisture, the hydrophilic sisal fiber swells. A higher cellulose content in the fiber further contributes to more water penetrating into the interface through the voids induced by the swelling of fibers. The neat M-C polyurethane samples also show water absorption behavior but with lower % than the biocomposites. This could be related to the microcellular nature of the synthesized polyurethane, as those micro voids would allow the water to diffuse into the polymer.

Water absorption tests show that alkali-treated M-C polyurethane sisal fiber biocomposites decrease the water uptake less than the untreated biocomposite under different fiber loadings (0–3 vol%). Alkali-treated fiber has fewer hydrophilic hydroxyl groups due to the removal of hemicellulose and lignin in the presence of 1.5% NaOH, which leads to a reduction of the water absorption.

### 2.3. SEM

**Figure 6:**
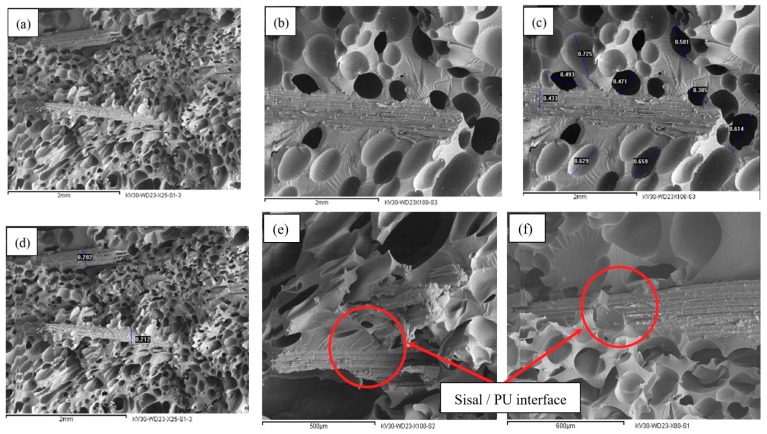
Surface morphology of M-C polyurethane /sisal biocomosites. (**a**) Untreated sisal, (**b**) alkali treatment, (**c**) PU foam cell size, (**d**) diameter of sisal fiber, (**e**) water-treated sisal/PU interface, (**f**) untreated sisal/PU interface.

Figure 6a–f shows good adhesion between the sisal fiber and the M-C PU matrix, which can be ascribed to the formation of bonds between the hydroxyl (–OH) groups of the fibers and the –NCO groups of the diisocyanate in urethane groups. 

Figure 6a,f show that untreated fibers are coated with more polyurethane layers compared with alkali- (Figure 6b) and water-treated (Figure 6d) sisal fiber, respectively. This indicates a better adhesion force between M-C PU and untreated sisal fiber than the NaOH-treated fiber. This agrees with the results obtained from the tensile tests. These results confirm the study findings that alkali treatment leads to decreased –OH groups in the hemicellulose contents, thus decreasing the probability of urethane formation among diisocyanates in the matrix and free hydroxyl groups in the sisal fiber. Figure 6c shows the microcellular structure of the polyurethane [42]. Closed homogeneously distributed cells are formed. They have average diameter of 0.55 mm. From Figure 6d, the average diameter of the sisal fibers is approximately 0.24 mm.

### 2.4. Viscoleasticity

**Figure 7:**
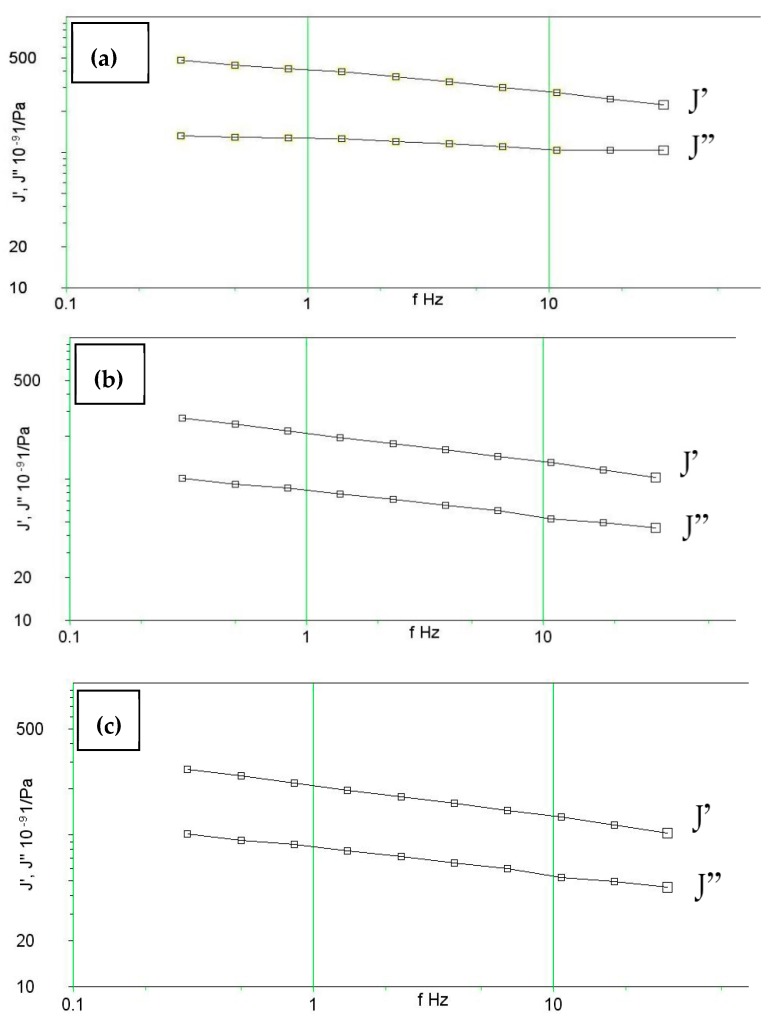
Viscoelastic behavior for 3% fiber composite at (**a**) 120 °C, (**b**) 100 °C and (**c**) 80 °C.

The effect of temperature on the viscoelastic behavior of the M-C polyurethane/sisal (1 vol%) biocomposite is illustrated in Figure 7. The real compliance J’ is always above the imaginary compliance J’’, which means that J’ is the dominant factor for describing the characteristics of the composite; thus, the material presents an elastic behavior rather than a viscous one. On decreasing the temperature from 120 to 80 °C, the values of J’ start decreasing, which indicates the transition of the material from a soft state to rigid state.

**Figure 8:**
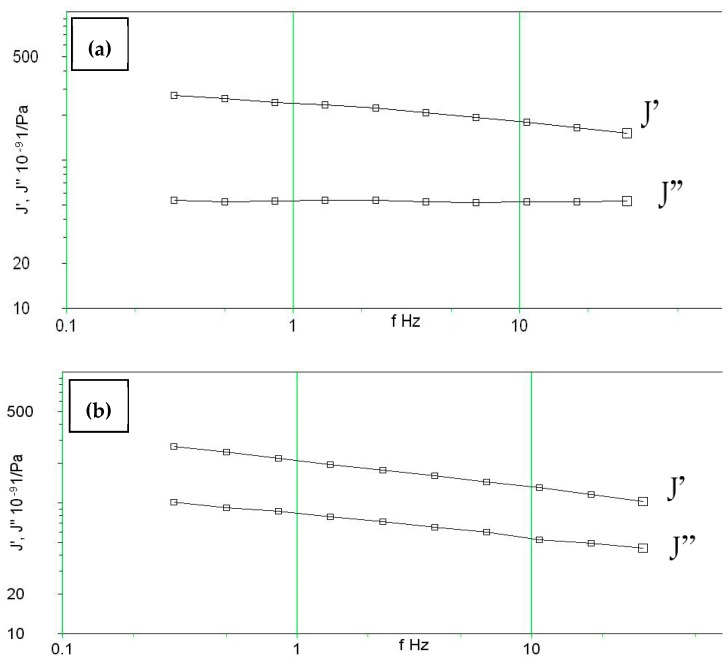
Viscoelastic behaviour of M-C PU/ sisal at 120 °C for (**a**) 1 vol% fiber composite (**b**) 3 vol%.

Figure 8a,b show the viscoelastic behavior of 3 and 1 vol% fiber at 120 °C, respectively. There is a significant decrease in the values of real compliance J’ (i.e., increase in storage modulus (G’) compared to those values of 1 vol% fibers at the same temperature. The composite exhibits more rigidity when the fibers are added, which is also a sign of the good interfacial adhesion between the fiber and the polymer matrix.

## 3. Materials and Methods

### 3.1. Materials

Polymeric methylene diphenyl diisocyanates (PMDI) with 33% isocyanate (–NCO) content were supplied by ELMOTAHEDA Co., (Tenth of Ramadan city, Egypt). Commercial castor oil with molecular weight 960 g/mole, and OH functionality 2.5 was purchased from A.S.O. Co. (Mumbai, India). An aluminum mold was manufactured and coated with a Teflon layer. Sisal fiber was supplied by The Agricultural Research Institute (Giza, Egypt). The chemical composition, physical, chemical and mechanical properties of the sisal fiber are listed in Table 2.

### 3.2. Mold Preparation

**Figure 9:**
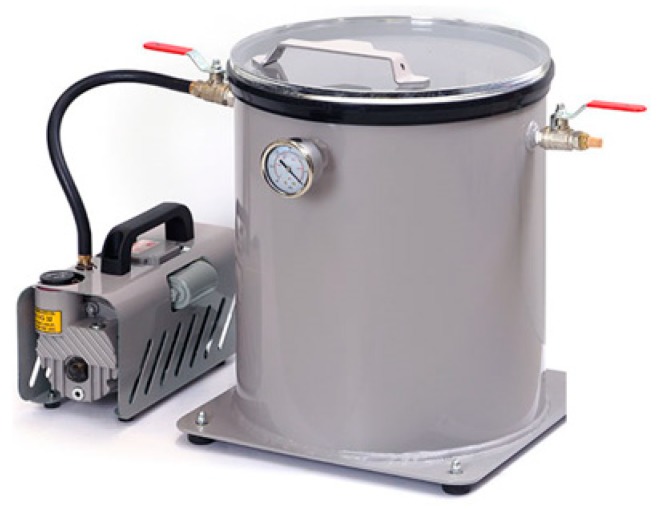
Vacuum system for castor oil degassing.

A mold with dimensions of 0.3 × 0.2 × 0.008 m^3^ (length × width × thickness) was prepared from an aluminum sheet. A hand mill was used to make a cavity with dimensions of 0.25 × 0.15 × 0.004 m^3^ (length × width × depth). Another aluminum sheet of 0.006 m thickness was used as the mold cover, allowing a clearance of 0.002 m between the mold and the cover. All the surfaces were ground to be smooth and coated with a layer of Teflon to avoid sticking of the polyurethane composites to the mold, as shown in Figure 9.

### 3.3. Castor Oil Treatment

Dehydration of castor oil was carried out to get rid of free and entrained water. Four liters of the oil were placed in a beaker and stirred using a magnetic stirrer for 5 h at 80 °C. The castor oil was placed in vacuum system as shown in Figure 9 for degassing and to avoid thermal degradation of the oil, which was then stored at room temperature (25 °C).

### 3.4. Sisal Treatment

Sisal fiber was cut into approximately 0.25 m pieces, and divided into three portions; one portion represented the untreated fiber, the second portion was treated with water, where the fiber was soaked in a basin containing distilled water for 24 h to remove impurities, dust and waste in order to enhance the effectiveness of fiber-matrix interfacial force. The third portion was treated by alkaline solution (NaOH 1.5% wt/wt). The soaking time was 24 h. The fiber was then removed from the basins and washed by distilled water to remove excess NaOH and then left to dry at ambient temperature (25 °C) for 24 h. Treatment of sisal fiber using diluted alkaline solution has been carried out to remove lignin and impurities which may help in improving sisal fiber-polymer interfacial bonding [43,44,45,46]. All sisal fiber sets were cut into equal lengths of 0.03 m. Finally, the three portions were heat-treated inside an oven at 100 °C for 24 h to get rid of any water content inside the fibers. They were then packed firmly in glass bottles to avoid exposure to air moisture.

### 3.5. Synthesis of Biocomposites

In this study, samples were synthesized with various parameters (temperature, stirring time, pressing time, percentage of defoaming agent and isocyanate to castor oil ratio) to set the conditions that would result in a suitable sample. It was noticed that low rates or periods of mixing led to precipitation of isocyanate forming a brown layer at the bottom of the matrix. Therefore, the mixing rate should be increased to 16.67 Hz for 15 min at least to enhance isocyanate distribution. The temperature of polymerization showed a high significant effect at high temperature (>100 °C); the samples were stiffer, which could be attributed to the forming of allophanate and biuret groups. The isocyanate to castor oil ratio significantly affected the stiffness of the samples [16]. A defoaming agent was used to control the size of the formed bubbles, making them homogeneously distributed. Before working on each sample, ethyl alcohol with concentration of 95% was used to clean the molds and ensure they were free of any impurities. Fifty mL of castor oil was introduced to a beaker, then 0.3 mL of defoaming agent was added and the mixture was stirred for 2 min using a mechanical mixer at 16.66 Hz. Next 35 mL of isocyanate was prepared and 7 mL of it were introduced every 2 min while stirring and the final mixture was stirred for 5 min. The required amount of sisal fibers (0, 1, 2 and 3 vol% for each of the untreated, water- and NaOH-treated fiber) was then added and mixed for 5 min using a mechanical stirrer. Higher fiber loadings showed agglomeration and less dispersibility during processing. The whole mixture was introduced to the mold in a viscous state in a zigzag way and was distributed along the mold using a glass rod. The mold was then heated at 120 °C for 5 min until the mixture started to solidify, then the mold was pressed at the same temperature for 10 min and plate samples with dimensions of 0.25 × 0.2 × 0.002–0.004 m^3^ (length × width × thickness) were finally released from the mold.

## 4. Tests

All samples were heated in an oven at 80 °C for 24 h for post-curing, then left for 5 days before performing any test, to ensure that they were completely crosslinked. The mean value of five mechanical and water uptake replicates tests are reported. Samples are cut from the same plates for mechanical, surface morphology, water uptake and viscoelastic testing.

### 4.1. Tensile Test

Tensile test was performed on a Z100 universal tensile machine (Zwick/Roell, Ulm, Germany). Test specimens were prepared according to ISO 527-4 for multidirectional fiber-reinforced materials. Specimens with dimensions of 0.2 × 0.25 × 0.002–0.004 m^3^ (length × width × thickness) were cut from the prepared plate samples having the different fiber volume fractions and treatment methods. Five specimens from each composite sample were tested and an average was calculated for the different values obtained from the stress-strain curve (elastic modulus, tensile strength and yield strength). Curves were then plotted to illustrate the effect of fiber loading (vol%) on mechanical properties. The secant method was used for measuring the elastic modulus, with stress ranging from 0.2 MPa to 10 MPa. Experimental results were then compared with the Cambridge Engineering Selector program (Granta Design, Cambridge, UK) results.

### 4.2. Water Uptake Test

The effect of fiber loadings (0, 0.5, 1, 2 and 3 vol%) and the different chemical treatments (untreated, water- and NaOH-treated) on the water uptake of the composites was investigated. Specimens broken from the tensile test were used to ensure the fibers were exposed to water. The initial weight of all the samples was first measured. Water uptake tests are conducted at 23 °C. Samples were immersed completely in water. Test was conducted according to DIN 53495 [47]. The samples were released and weighed every 60 min for the first 2 h, then weighed every hour for four hours. A fine tissue was used to remove surface water droplets. Samples are weighed and returned into the water bath within 60 s.

### 4.3. Viscoelasticity Test

The instrument used for measuring the visco-elastic properties was a Bohlin Gemini 200 Rheometer from Malvern (Worcestershire, UK). The rheometer has a torque range of 0.05 × 10^−6^ to 0.2 Nm, a torque resolution higher than 1 × 10 ^−9^ Nm, position resolution of 50 nano radians, frequency range of 1 × 10^−6^ to 100 Hz. Normal force measurement range of 0.001 to 20 N and temperature range of −150 °C to 550 °C. In the Gemini rheometer, the sample was placed in the form of a disc between two parallel metal plates, where the lower one was fixed, and the upper one rotated by the instrument (usually as periodic oscillations as the sample was solid). The rotation angle was measured by the instrument and from the data, the deformation angles γ (= the strain) were calculated, and an average value was determined. The angular momentum was measured by electrical force transducers. Accordingly, the shear stress τ has been determined. Samples used in this test were cut from the composite plates containing untreated fiber with volume fractions of 0.1 and 3% in a disc form with diameter of 40 mm, similar to the plates between which the samples were placed. A frequency range of 0.3–30 Hz was applied and each sample was tested at temperatures of 80, 100 and 120 °C.

### 4.4. Scanning Electron Microscopy (SEM)

A S150 A gold sputter coater from Edwards (Crowley, UK) was used to coat the mounted specimens with gold before they entered the SEM. Samples were kept inside it for 6 min under vacuum (4 × 10^−1^ bar) at 40 mA. If the specimen was not finely covered with an electron-opaque substance like gold, the electron beam would travel right through the specimen, creating no image and perhaps even destroying the sample. The electron microscope used was Inca x-sight from Oxford Instruments (Wycombe, UK) working at 30 kV. The work distance for the samples was 23 mm. The microscope was connected to a JXA-840A electron probe microanalyzer from JEOL (Tokyo, Japan), on which the images were shown and adjusted. Computer software was used to save the images and measure different lengths on the images. The magnification of each image is reported on it.

## 5. Conclusions

The tensile tests showed that mechanical properties of M-C polyurethane sisal biocomposites generally increased with increasing fiber loading. Untreated sisal fiber showed the highest improvements in elastic, yield and tensile strength; while the alkali-treated sisal fiber showed the least improvements, with 59.17%, 13.27% and 7.1% increases in the elastic modulus, tensile and yield strengths, respectively. This is probably due to the attack on the fiber –OH groups react with the NCO groups of the isocyanate by NaOH, which consequently decreases the interfacial adhesion. The SEM results showed good agreement with the mechanical tests. CES simulation results showed that thermal expansion of M-C PU is remarkably improved in the presence of sisal fiber. Less water absorption can be obtained when sisal fibers are treated with alkaline solution. Visoelastic measurements showed that the composites exhibit an elastic trend as measured by the real compliance (J’) values. Moreover, increasing the fiber loadings results in increasing storage modulus G’), which means that the samples become more rigid. Applying temperatures of 120, 100 and 80 °C, respectively, led to an increase in stiffness at different frequencies. M-C polyurethane sisal biocomposites can be used in several applications such as thermal and electrical insulation, automotive interior parts, furniture, packing and biomedical uses.

## Figures and Tables

**Table 1:** Thermal and mechanical properties of M-C polyurethane (PU) and M-C PU/sisal biocomposites.

	M-C PU/Sisal (Random Short Fiber Composite) 1%vf	M-C PU/Sisal (Random Short Fiber Composite) 1.26%vf	M-C PU/Sisal (Random Short Fiber Composite) 1.59%vf	M-C PU/Sisal (Random Short Fiber Composite) 2%vf	M-C PU/Sisal (Random Short Fiber Composite) 2.52%vf	M-C PU/Sisal (Random Short Fiber Composite) 3.17%vf	M-C PU/Sisal (Random Short Fiber Composite) 4%vf	M-C PU
Density (kg/m^3^)	607	610	613	616	621	626	634	599
Young’s modulus (GPa)	0.521	0.539	0.562	0.59	0.626	0.67	0.726	0.43
Yield strength (elastic limit) (MPa)	6.7	7.09	7.59	8.21	8.99	9.96	11.2	5.1
Thermal expansion coefficient (µstrain/°C)	90.5	86.1	81.4	76.3	70.9	65.4	60	114

**Table 2:** Chemical composition and properties of sisal fiber.

**Chemical Composition**	
Cellulose (%)	41.6–62.6
Hemi cellulose (%)	9.2–14.6
Lignin (%)	11.4–19.5
**Mechanical Properties**	
Young’s modulus (GPa)	9.4–22
Yield strength (elastic limit) (GPa)	460–576
Tensile strength (MPa)	511–640
Elongation strain (%)	2–7
Flexural modulus (GPa)	9.4–22
Fatigue strength at 10^7^ cycles (MPa)	220–316
Mechanical loss coefficient (tan delta)	0.00407–0.00753
**Thermal Properties**	
Glass temperature (°C)	380–390
Maximum service temperature (°C)	400–420
Thermal conductivity (W/m °C)	0.25–0.35
Specific heat capacity (J/Kg °C)	1.2 ×10 3- 1.22×103
**Absorption & Permeability**	
Water absorption @ 24 h (%)	2–2.4
**Durability**	
Water (salt)	Excellent
Weak acids	Acceptable
Weak alkalis	Acceptable
Organic solvents	Acceptable
UV radiation (sunlight)	Good
**Physical Properties**	
Density (kg/m^3^)	1410
Fiber diameter(µm)	145–440
